# Sex-dependent effects of chronic intermittent hypoxia: Implication for obstructive sleep apnea

**DOI:** 10.21203/rs.3.rs-3898670/v1

**Published:** 2024-01-29

**Authors:** Steve Mabry, Jessica L. Bradshaw, Jennifer J. Gardner, E. Nicole Wilson, Rebecca Cunningham

## Abstract

Background Obstructive sleep apnea (OSA) affects 10–26% of adults in the United States with known sex differences in prevalence and severity. OSA is characterized by elevated inflammation, oxidative stress (OS), and cognitive dysfunction. However, there is a paucity of data regarding the role of sex in the OSA phenotype. Prior findings suggest women exhibit different OSA phenotypes than men, which could result in under-reported OSA prevalence in women. To examine the relationship between OSA and sex, we used chronic intermittent hypoxia (CIH) to model OSA in rats. We hypothesized that CIH would produce sex-dependent phenotypes of inflammation, OS, and cognitive dysfunction, and these sex differences would be dependent on mitochondrial oxidative stress (mtOS). Methods Adult male and female Sprague Dawley rats were exposed to CIH or normoxia for 14 days to examine the impact of sex on CIH-associated circulating inflammation (IL-1β, IL-4, IL-6, IL-10, TNF-α), circulating OS, and behavior (recollective and spatial memory; gross and fine motor function; anxiety-like behaviors; and compulsive behaviors). A subset of rats was implanted with osmotic minipumps containing either a mitochondria-targeting antioxidant (MitoTEMPOL) or saline vehicle 1 week prior to CIH initiation to examine how inhibiting mtOS would affect the CIH phenotype. Results Sex-specific differences in CIH-induced inflammation, OS, motor function, and compulsive behavior were observed. In female rats, CIH increased inflammation (plasma IL-6 and IL-6/IL-10 ratio) and impaired fine motor function. Conversely, CIH elevated circulating OS and compulsivity in males. These sex-dependent effects of CIH were blocked by inhibiting mtOS. Interestingly, CIH impaired recollective memory in both sexes but these effects were not mediated by mtOS. No effects of CIH were observed on spatial memory, gross motor function, or anxiety-like behavior, regardless of sex. Conclusions Our results indicate that the impact of CIH is dependent on sex, such as an inflammatory response and OS response in females and males, respectively, that are mediated by mtOS. Interestingly, there was no effect of sex or mtOS in CIH-induced impairment of recollective memory. These results indicate that mtOS is involved in the sex differences observed in CIH, but a different mechanism underlies CIH-induced memory impairments.

### Plain Language Summary

Sleep apnea is a common sleeping condition in adults with a wide range of symptoms that include inflammation, oxidative stress, memory problems, anxiety, and compulsivity. Men are diagnosed with sleep apnea more often than women. Although there is limited information on how sleep apnea affects men and women differently, previous studies suggest that women may exhibit different sleep apnea symptoms than men. To examine the impact of male and female sex on common sleep apnea symptoms, we exposed adult male and female rats to a model of sleep apnea called chronic intermittent hypoxia (CIH). We found that many effects of CIH were different in males and females. CIH females had increased inflammation and motor problems, whereas CIH males had increased oxidative stress and compulsivity. To investigate the reason for these CIH sex differences, we blocked mitochondrial oxidative stress. Blocking mitochondrial oxidative stress decreased CIH associated sex differences. However, blocking mitochondrial oxidative stress had no impact on CIH-induced memory impairment that was observed in male and female rats. Our findings support previous reports that suggest that women exhibit different sleep apnea symptoms than men. Further, we extend these findings by showing that mitochondrial oxidative stress is involved in these sex differences. Clinically, patients diagnosed with sleep apnea are typically treated with continuous positive airway pressure (CPAP) machines, which have high rates of non-compliance (15-40%). Therefore, understanding why sleep apnea is causing these symptoms will be important in developing therapeutics.

### Highlights

- This is the first study to examine the contribution of sex on CIH-induced inflammation, oxidative stress, and behavioral dysfunction in rats.
- CIH increased circulating inflammation and fine motor dysfunction in females, whereas CIH increased circulating oxidative stress and compulsivity in males.
- Inhibition of mitochondrial oxidative stress blocked sex-specific effects of CIH in males and females.
- CIH induced recollective memory impairments in males and females, which was not prevented by blocking mitochondrial oxidative stress.

### Background

Obstructive sleep apnea (OSA) is a highly prevalent sleeping disorder which is observed to affect between 10–26% of adults [1–3]. Men are 2–4 times more likely to be diagnosed with OSA than women [1–3]. However, the prevalence of OSA may be under-reported for women, as OSA symptoms are different in women compared to men [4]. Women are more likely to present with atypical symptoms of OSA, even in mild OSA, that is associated with lower levels of apnea-hypopnea index (AHI) scores (hypoxic events per hour of sleeping) than men [4, 5]. Symptoms of OSA include cognitive dysfunction, anxiety, and compulsivity [6–12], along with increased inflammation and oxidative stress (OS) [13–18]. Though women have been observed to have greater risk for OSA-associated cognitive dysfunction [3, 19], the role of sex on OSA symptoms is not well-known.

Patients diagnosed with OSA are typically treated with continuous positive airway pressure (CPAP) machines, the gold standard for therapy [20–22]. Effective CPAP therapy can reduce inflammation, OS, and cognitive dysfunction [21, 23–25]. However, adherence to CPAP therapy is a major issue, as 15–40% of patients are non-compliant with prescribed machine usage [20–22]. Even patients that are adherent to CPAP therapy may not be receiving proper treatment, due to anatomical variations or ineffective machine air pressure titration [20, 22]. This lack of efficacy has led to increased research to determine OSA mechanisms with the goal of developing conjunctive pharmaceutical therapies for OSA [26–28]. Current drugs that are approved for OSA treatment are focused on symptom therapy, such as reducing daytime sleepiness and improving attention [27, 28] rather than OSA pathophysiology as a result of OSA hypoxia.

Hypoxia induced by OSA is observed to increase many circulating markers of OS and inflammation [13–18]. This can induce a feed-forward cycle, in which chronic inflammation increases OS [29, 30], and chronic OS can increase inflammation [31, 32]. In addition, sex also plays a role in OS and inflammation [33, 34]. Women exhibit higher baseline levels of OS compared to men [35–37]. Furthermore, women experience greater levels of inflammation and autoimmunity compared to men [38–40]. Based on these findings, sex differences observed in OSA may be related to basal sex differences in inflammatory and OS status. Since the mitochondria are one of the primary generators of OS within the cell [41, 42] and mitochondrial dysfunction has been observed in OSA [43–45], mitochondrial OS (mtOS) may mediate sex differences in OSA.

To examine the impact of sex on OSA-associated symptoms (inflammation, OS, memory, motor, anxiety, compulsivity), we utilized an experimental rat model of OSA called chronic intermittent hypoxia (CIH). CIH exposure replicates the fragmented episodes of hypoxia observed in OSA [46]. Further, to examine the mechanisms of mtOS on sex differences in CIH, we implanted osmotic pumps subcutaneously in a subgroup of rats to continuously administer a mitochondria-targeting superoxide dismutase mimetic, MitoTEMPOL (MT), to block mtOS. The inflammatory, OS, and behavioral phenotypes of CIH in males are clearly defined. CIH increases inflammation [6, 47, 48], increases OS [6, 49, 50], impairs recollective memory [51–53], impairs spatial learning and memory [47, 54–56], and increases anxiety-like behavior [51, 52, 57, 58]. Studies examining sex as a biological variable in CIH are scarce. Most of these studies have been conducted only in mice, showing CIH increased circulating OS in male but not female mice [49] and impaired spatial learning and memory in ovariectomized female mice but not intact females [59]. Given the limited data on the effects of CIH in females, we predicted that CIH would produce sex-dependent increases in inflammation and OS, alongside cognitive dysfunction. Further, we predicted that inhibiting mtOS would prevent the CIH phenotype of increased inflammation, increased OS, and cognitive dysfunction.

## Methods

## Animals

All experiments were conducted using adult virgin Sprague Dawley male and female rats (aged 3–4 months, Charles River, Wilmington, MA). Male and female rats were housed in separate rooms in our animal facility on a 12-hour (hr) reverse light cycle (lights were off at 09:00). Reverse lighting allowed behavioral testing to be conducted during the active phase of the circadian cycle. Food and water were provided *ad libitum*. Rats (n = 7–13/group) were randomly assigned to either normoxia (room air) or CIH treatment conditions. A subgroup of rats (n = 6–10) were given MT. This resulted in four groups/sex: Normoxic Vehicle, Normoxic MT, CIH Vehicle, CIH MT. To acclimatize the rats to operator handling and reduce stress responses during behavior testing, rats were handled daily, beginning one week prior to the start of behavior testing. At the conclusion of behavior testing, the rats were anesthetized with 2–3% isoflurane and euthanized via decapitation during the active phase of the circadian cycle (09:00–11:00). All experiments were conducted in agreement with the Guide for the Care and Use of Laboratory Animals of the National Institutes of Health and the ARRIVE guidelines. These protocols were approved by the Institutional Animal Care and Use Committee of the University of North Texas Health Science Center.

## Osmotic minipump implantation

One week prior to the initiation of CIH, all rats were instrumented with an osmotic minipump (Alzet Mini-Osmotic Pump Model 2002, Durect Corporation, Cupertino, CA) implanted subcutaneously between the scapula [60]. Minipumps contained either 0.9% saline vehicle or MitoTEMPOL (0.7 mg/kg/day; Caymen Chemical Company, Ann Arbor, MI; MT) dissolved in saline. MT combines the antioxidant moiety TEMPOL, with a lipophilic cation triphenylphosphonium [61, 62]. Triphenylphosphonium increases mitochondrial aggregation of MT by several hundred-fold over TEMPOL alone [62–64]. MT has been observed to reduce mtOS *in vitro* and *in vivo* [64, 65]. Drugs like MT are easily administered with multiple biologically active routes of administration and high blood-brain barrier permeability [66–68]. The dose of MT was chosen based on previous *in vivo* studies [65, 69, 70]. To ensure proper osmotic function as indicated by bubbling present on the surface of pumps, all pumps were incubated in a 37°C water bath for at least 24 hours prior to implantation. Surgeries were performed using aseptic techniques with isoflurane (2–3%) anesthetic.

## Chronic intermittent hypoxia protocol

One week prior to the initiation of the CIH protocol, the home cages (clear plastic containers) of the rats were placed into Oxycycler chambers (76.2 x 50.8 x 50.8cm, BioSpherix, Lacona, NY, USA) to acclimatize the rats to the chambers under normoxic conditions. During their sleep phase of the circadian cycle, CIH was performed for 8 hrs starting at 21:00. The CIH protocol consisted of intermittent oxygen reduction from 21% (room air) to 10% in 6-minute cycles per hour (i.e., 10 cycles/hr) over 8 hrs/day for a period of 14 days, as previously described [6, 46, 71, 72]. 10 CIH cycles per hour results in an AHI of 10, which is consistent with mild sleep apnea in humans [25, 72].

## Behavioral Tasks

Behavioral studies were conducted between days 8 and 14 of CIH from 09:45 to 17:00 during the active phase of the circadian cycle. The order of the behavior tests was randomized. Male and female rats were behaviorally tested in separate cohorts to prevent potential confounding effects of pheromones on behavior [73, 74]. All testing equipment (e.g., marbles, arenas, tanks) were thoroughly cleaned with 70% ethanol between each rat. All behavior studies were conducted under red lighting and recorded for later analysis by an investigator blinded to treatment groups. Behavior tests were used to assess compulsive behaviors (marble burying test) [75, 76], fine motor function (footfalls, rearing behavior) [50], anxiety-like behaviors (center entries and center duration in an open field) [77, 78], spatial learning and memory (Morris water maze) [77, 79], and recollective memory (novel object) [80, 81]. To avoid a learning confound due to battery testing (e.g., open field tests [82–84] and Morris water maze [82, 83]), one behavioral task per index of interest was performed.

## Modified open field: Fine motor function

Fine motor function was assessed using a small novel open field arena (40.64 x 40.64 x 38.1 cm) with a bi-directional main field bar (San Diego Instruments Photobeam Activity System; Open Field Arena), as previously published [46, 50, 71]. To increase the difficulty of locomotion, a wire mesh platform elevated 2 cm above the floor was placed in the arena. The rats were allowed 10 minutes to explore the arena. Fine motor function was classified by distance traveled, rearing behavior (assisted, unassisted, total), and footfalls past the elevated wire mesh. Rears were separated into assisted or unassisted when the animal stood on its hindlimbs with (assisted) or without (unassisted) its forelimbs bracing against the wall [46].

## Open field: Gross motor function and anxiety-like behavior

Using a large novel open field arena (60.96 x 60.9 x 38.1 cm), we assessed gross motor function and anxiety-like behavior during a 5-minute trial. Behaviors were recorded using ANY-maze software (v. 5.14, Stoelting CO.). This open field duration has been previously observed to be sensitive to differences in anxiety-like behavior and stress in rats [85–87]. Gross motor function was examined by distance traveled in the open field. Anxiety-like behaviors were tracked by number of entries (frequency) to the center of the field and time spent (duration) within the center of the open field [50, 71]. In addition, this task allowed habituation to the large open field arena in order to conduct the novel object recognition task.

## Novel object recognition task: Recollective memory

Following habituation to the open field test using the large arena (60.96 x 60.9 x 38.1 cm), we conducted the novel object recognition task [50]. In this task, two identical objects (building blocks) were placed in adjacent corners of the arena, and the rats were given 5 minutes to interact with the objects and then removed from the arena. One hr later, one of the objects was replaced with a novel object (spherical toy ball), and the rats were given 3 minutes to explore the arena and interact with the objects. Contacts with the novel object and latency to the novel object were recorded as measures of short-term recollective memory retention [50, 80, 81].

## Morris water maze: Spatial learning and memory

To examine spatial memory, the Morris water maze test was used according to our published protocols [50, 71]. Behaviors were recorded using ANY-maze software (v. 5.14, Stoelting Co.). Over a period of 4 days, rats were trained to find a submerged platform in a pool filled with opaque water (23–25°C) and remain on the platform for 20 seconds until removed by the operator. Day 1 of training consisted of visible platform pre-training where the platform was moved on each trial (3/day). On days 2–4, the platform was submerged for spatial training and remained in the same position for all trials. An unweighted learning index (LI) and a weighted learning index (WLI) were generated using the latency data from days 2, 3, and 4 of spatial training. The unweighted LI was generated as the average latency to the target of all trials of all training days [71, 88]. The WLI was calculated using the average of normoxic control rats/sex [88, 89]. Specifically, WLI is calculated by obtaining a multiplier based on initial performance on day 2: day 2 multiplier ($\bar{X}_{\text{Day 2 Control Latency}}/\bar{X}_{\text{Day 2 Control Latency}}$); day 3 multiplier ($\bar{X}_{\text{Day 2 Control Latency}}/\bar{X}_{\text{Day 3 Control Latency}}$); and day 4 multiplier ($\bar{X}_{\text{Day 2 Control Latency}}/\bar{X}_{\text{Day 4 Control Latency}}$). Then, all latencies for each day were multiplied by their respective multiplier (day 2 latency*day 2 multiplier; day 3 latency*day 3 multiplier; day 4 latency*day 4 multiplier) to generate a weighted latency score (WLS). The final WLI used for analysis was the sum total of each WLS over days 2, 3 and 4 for each rat. The unweighted LI and WLI scores were used as indicators of spatial learning where lower unweighted LI and WLI scores indicated greater spatial learning ability [88, 89]. Spatial memory was assessed following 4 days of training, in which each rat was administered a probe trial (the underwater platform was removed). Latency and pathlength to the target during the probe trial were used as indicators of spatial memory.

## Marble burying test: Compulsive behavior

To examine compulsive behaviors, the marble burying test was conducted following published protocols [71, 75, 76]. To conduct this test, the floor of the testing arena (50 x 25 x 30 cm) was thoroughly covered with 1 cm of rodent bedding litter to allow the rats to easily bury marbles. Twenty marbles of similar color and size (1.5 cm) were spaced evenly in a 4 x 5 grid on one side of the arena base. Each rat was given 10 minutes to explore and interact with the marbles. Operators visually monitored the experiment and manually recorded behaviors. After 10 minutes, the rat was removed and the number of marbles buried (75% or more) was photographed and quantified.

## Sample collection

At the conclusion of behavior tests, rats were anesthetized with isoflurane (2–3%) and decapitated during the first 2 hrs of the rats' active phase of the circadian rhythm to collect tissue and plasma samples [6, 46, 50, 71]. Trunk blood was collected in EDTA tubes, and then centrifuged at 2,000 x g for 10 minutes at 4°C to collect plasma. Plasma was stored at -80°C until assayed for circulating inflammation and oxidized proteins.

## High throughput multiplex inflammation panel

A MILLIPLEX® rat cytokine/chemokine magnetic bead panel (Sigma Millipore, Cat # RECYTMAG-65K) utilizing antibodies against IL-1β, IL-4, IL-6, IL-10, and TNF-α was used to quantify circulating inflammatory cytokines. IL-6, TNF-α, and IL-1β were selected as common pro-inflammatory cytokines

[90–92], while IL-10 and IL-4 were selected as common anti-inflammatory cytokines [93, 94]. An 18-hr overnight incubation was performed according to manufacturer instructions. All samples were diluted 1:2 in assay buffer prior to running the assay according to manufacturer's instructions. Samples were run in duplicate and cytokines were measured on Luminex® 200™ using xPONENT® software version 4.3 (Luminex Corporation, Austin, TX). Quality control values for each cytokine were within the range provided by the manufacturer. The ratio of IL-6/IL-10 was used to investigate the overall relationship between pro- and anti-inflammatory cytokines [95]. Due to limited plasma availability, no analyses of normoxic MT plasma were performed.

### Advanced oxidation protein products assay

Circulating plasma OS was assayed using OxiSelect Advanced Oxidation Protein Products (AOPP) kit (Cell Biolabs, Inc., San Diego, CA) according to our previously published protocols [6, 46]. The micromolar (µM) concentration of oxidized proteins in the plasma were measured relative to a known standard. Chloramine in the kit reacts with oxidized proteins to produce a color change, which is read at 340 nm. To account for colorimetric interference from the plasma samples, a background correction was performed for all samples. All samples were diluted 1:2 in assay buffer for analysis.

### Statistical analysis

Statistical analyses were conducted in IBM® SPSS® (SPSS® v. 29.0.0, IBM®, 2022). Normality of data distribution was tested using the Shapiro-Wilk test. Data with non-Gaussian distribution were normalized by log base 10 transformation (x = lg10(x)) or by square root transformation (x = sqrt(x)) through the SPSS® transformation function. Outliers greater than 2 standard deviations from the mean were removed from analysis. 3-way ANOVAs were conducted using the factors of CIH, drug treatment, and sex, with the exception of 2-way ANOVAs used for inflammatory cytokine analyses. For all analyses, we provide the F values, degrees of freedom, p-values, and $\eta^2$ (measure of effect size). Following ANOVA testing, a Fisher's LSD post-hoc test was used to determine specific group differences for significant results. Results are presented as mean ± S.E.M. unless otherwise indicated. Significance was defined as $p \leq 0.05$. We report non-significant trends as $p \leq 0.055$. Post-hoc significance is indicated in figures and tables by unique letters ($p \leq 0.05$), in which shared letters are not significantly different from each other.

## Results

#### CIH induced a pro-inflammatory state in female rats that was reduced by blocking mitochondrial oxidative stress.

To examine the impact of sex and CIH on inflammatory cytokines, we used 2-way ANOVAs with the factors of sex and CIH in vehicle rats. We observed a significant interaction between CIH and sex on IL-6 levels ($F_{1, 19}$=8.420; p = 0.009; $\eta^2$ = 0.304; Fig. 1A). Specifically, CIH increased IL-6 levels only in female rats, and normoxic males had higher IL-6 levels than normoxic females. There was no observed effect of CIH or sex on IL-10 levels in vehicle rats (Table 1). However, there was a significant interaction between

CIH and sex on the IL-6/IL-10 ratio, wherein CIH increased the IL-6/IL-10 ratio in female rats ($F_{1, 19}$=15.683; p < 0.001; $\eta^2$ = 0.403; Fig. 1B). No effects of CIH or sex were observed on TNF-α, IL-1β, or IL-4 levels in vehicle rats (Table 1).

Table 1
Inflammatory cytokines in vehicle rats

| Vehicle | | | |
|---|---|---|---|
| | | **Normoxia** | **CIH** |
| **TNF-α (pg/mL)** | **Female** | 4.94 ± 1.71 | 6.60 ± 4.74 |
| | **Male** | 4.78 ± 0.74 | 4.82 ± 4.13 |
| **IL-1β (pg/mL)** | **Female** | 90.02 ± 169.54 | 11.73 ± 13.81 |
| | **Male** | 89.61 ± 137.64 | 64.40 ± 82.56 |
| **IL-10 (pg/mL)** | **Female** | 377.89 ± 559.37 | 79.79 ± 130.46 |
| | **Male** | 416.12 ± 565.47 | 378.63 ± 434.11 |
| **IL-4 (pg/mL)** | **Female** | 54.01 ± 50.48 | 99.02 ± 174.22 |
| | **Male** | 60.45 ± 65.50 | 15.87 ± 22.19 |

Plasma concentrations for pro-inflammatory cytokines TNF-α and IL-1β and anti-inflammatory cytokines IL-10 and IL-4. All cytokines plasma concentrations are reported in pg/mL. All values presented as mean ± SD. Data for inflammatory cytokines was log transformed for analysis. Analyzed by 2-way ANOVA with Fisher's LSD multiple comparisons tests, n = 5–6/group. *CIH: Chronic intermittent hypoxia.*

To examine the impact of sex and mtOS on CIH-induced inflammation, we used 2-way ANOVAs with the factors of sex and MT treatment (vehicle or MT) in CIH exposed rats. We observed that CIH females had higher IL-6 levels than CIH males ($F_{1, 20}$=5.327; p = 0.032; $\eta^2$ = 0.210; Fig. 2A). Inhibition of mtOS with MT had no effect on CIH-elevated IL-6 levels (Fig. 2A). Similar to IL-6, CIH female rats had higher TNF-α levels than CIH males, regardless of MT ($F_{1, 18}$=8.406; p = 0.010; $\eta^2$ = 0.318; Fig. 2B). There was a significant interaction between sex and MT on IL-1β levels in CIH rats, where vehicle males had higher IL-1β levels than vehicle females, and MT males had lower IL-1β levels than vehicle males and MT females ($F_{1, 19}$=7.844; p = 0.011; $\eta^2$ = 0.287; Fig. 2C). A significant interaction was observed between sex and MT on IL-10 levels in CIH rats, where vehicle males had higher IL-10 levels than vehicle females, and MT increased IL-10 levels only in females ($F_{1, 20}$=7.077; p = 0.015; $\eta^2$ = 0.237; Fig. 2D). We observed a main effect of sex on the IL-6/IL-10 ratio in CIH rats, where CIH-exposed females showed higher IL-6/IL-10 ratios compared to CIH-exposed males ($F_{1, 20}$=8.331; p = 0.009; $\eta^2$ = 0.237; Fig. 2E). We also observed a significant interaction between sex and MT on the IL-6/IL-10 ratio in CIH rats, where MT only reduced the IL-6/IL-10 ratio in females ($F_{1, 20}$=5.817; p = 0.026; $\eta^2$ = 0.165; Fig. 2E). No effects of sex or MT were observed on IL-4 levels in CIH rats (Table 2).

Table 2
Inflammatory cytokines in CIH rats

| CIH | | | |
|---|---|---|---|
| | | **Vehicle** | **MT** |
| **IL-4 (pg/mL)** | **Female** | 99.02 ± 174.22 | 94.07 ± 88.18 |
| | **Male** | 15.87 ± 22.19 | 82.95 ± 84.45 |

Plasma concentrations for anti-inflammatory cytokine IL-4. IL-4 plasma concentrations reported in pg/mL. All values presented as mean ± SD. Data for inflammatory cytokines was log transformed for analysis. Analyzed by 2-way ANOVA with Fisher's LSD multiple comparisons tests, n = 6/group. *CIH: Chronic intermittent hypoxia; MT: MitoTEMPOL.*

**CIH increased circulating oxidative stress in males that was reduced by blocking mitochondrial oxidative stress.**

We observed that circulating OS (plasma oxidized proteins; AOPP) was dependent on CIH, sex, and inhibition of mtOS with MT: main effects of CIH ($F_{1, 46}$=4.071; p = 0.049; $\eta^2$ = 0.040), sex ($F_{1, 46}$=18.743; p < 0.001; $\eta^2$ = 0.184), and MT were observed on AOPP ($F_{1, 46}$=19.286; p < 0.001; $\eta^2$ = 0.190; Fig. 3). We also observed significant interactions between CIH and sex ($F_{1, 46}$=6.432; p = 0.015; $\eta^2$ = 0.063) and between CIH, sex, and MT on AOPP ($F_{1, 46}$=6.590; p = 0.014; $\eta^2$ = 0.065; Fig. 3). Overall, AOPP concentrations were higher in females than males (p ≤ 0.05). Normoxic females had the highest AOPP concentrations, which were reduced by MT (p ≤ 0.05). Normoxic MT males had the lowest AOPP concentrations, which were significantly lower than all females (p ≤ 0.05). Exposure to CIH exhibited sex-dependent effects. CIH reduced AOPP concentrations in females (p ≤ 0.05), and inhibition of mtOS using MT only reduced AOPP concentrations in normoxic females with no effect in CIH females. In contrast to females, CIH increased AOPP concentrations in vehicle males (p ≤ 0.05), and this CIH-induced OS was blocked by inhibiting mtOS with MT. AOPP concentrations in CIH vehicle males were increased to levels observed in females (except MT normoxic females) (p ≤ 0.05).

**CIH impaired recollective memory in male and female rats, which was unaffected by inhibiting mitochondrial oxidative stress.**

Recollective memory was measured in the novel object recognition task. CIH significantly increased latency to the novel object ($F_{1, 60}$=14.011; p < 0.001; $\eta^2$ = 0.141), and this memory impairment was unaffected by blocking mtOS with MT (Fig. 4A). Females also had shorter latency to the novel object than males ($F_{1, 60}$=4.386; p = 0.040; $\eta^2$ = 0.053; Fig. 4A), especially in normoxic females compared to CIH males (p ≤ 0.05). In addition to increasing latency to the novel object, CIH significantly decreased novel object contacts ($F_{1, 60}$=9.664; p = 0.003; $\eta^2$ = 0.131; Fig. 4B) with the greatest effect seen in vehicle females and MT males (p ≤ 0.05). No effects of sex or inhibiting mtOS with MT were observed on novel object contacts (Fig. 4B).

We also examined spatial learning and memory using the Morris water maze. We observed lower WLI in female rats compared to males ($F_{1, 49}$=8.385; p = 0.006; $\eta^2$ = 0.144), regardless of CIH or inhibition of mtOS with MT (Fig. 5A). No effects of CIH, sex, or MT were observed on unweighted LI (Fig. 5B). Next, we examined spatial memory by performing a probe trial test on the final day of the Morris water maze. In contrast to the WLI scores observed, males exhibited a trend towards shorter latency to the probe target in Morris water maze than females ($F_{1, 52}$=3.905; p = 0.053; $\eta^2$ = 0.067; Fig. 5C). No effects of CIH or inhibition of mtOS with MT were observed on latency to the probe target. We observed no effects of CIH, sex or mtOS inhibition on pathlength to the probe target in the Morris water maze (Table 3).

Table 3
Morris water maze pathlength to target

| **Normoxia** | | | | **CIH** | | | |
|---|---|---|---|---|---|---|---|
| | | **Vehicle** | **MT** | | | **Vehicle** | **MT** |
| **Pathlength to Target (cm)** | **Female** | 561.50 ± 136.63 | 646.77 ± 169.81 | **Pathlength to Target (cm)** | **Female** | 644.33 ± 133.69 | 716.73 ± 99.12 |
| | **Male** | 682.71 ± 149.04 | 630.01 ± 128.56 | | **Male** | 651.58 ± 131.39 | 608.37 ± 111.85 |

Pathlength (cm) for rats to find the target location during the probe trial. All values presented as mean ± SD. Analyzed by 3-way ANOVA with Fisher's LSD multiple comparisons tests, n=5-8/group. *CIH: Chronic intermittent hypoxia; MT: MitoTEMPOL.*

**CIH impaired fine motor function in female rats that was reduced by blocking mitochondrial oxidative stress.**

To examine gross motor function, we used a large novel open field (60.96 x 60.9 x 38.1 cm). We observed that female rats traveled farther than male rats ($F_{1, 60}$=10.598; p = 0.002; $\eta^2$ = 0.136; Fig. 6A), particularly compared to CIH males with MT treatment ($p \leq 0.05$). No effects of CIH or mtOS inhibition with MT were observed on distance traveled in the open field (Fig. 6A).

Next, we examined fine motor function using a novel modified open field (MOF; 40.64 x 40.64 x 38.1 cm) in which a wire mesh platform elevated 2 cm above the floor was placed in the arena. We observed a significant effect of CIH on MOF distance traveled ($F_{1, 64}$=4.706; p = 0.034; $\eta^2$ = 0.058; Fig. 6B). In addition, we observed a significant interaction between CIH, sex, and mtOS with MT on distance traveled in the MOF ($F_{1, 64}$=6.683; p = 0.012; $\eta^2$ = 0.083; Fig. 6B). Specifically, CIH reduced MOF distance traveled in vehicle females, which was blocked by mtOS inhibition using MT ($p \leq 0.05$). CIH did not impact fine motor function (distance traveled) in males. However, inhibition of mtOS did increase fine motor function in normoxic males ($p \leq 0.05$). No effects of CIH, sex, or MT, were observed on other measures of fine motor function - assisted rears, unassisted rears, total rears, or footfalls in a modified open field (Table 4).

Table 4
Fine motor behavior in modified open field

| Normoxia | | | | CIH | | | |
|---|---|---|---|---|---|---|---|
| | | **Vehicle** | **MT** | | | **Vehicle** | **MT** |
| **Assisted Rears** | **Female** | 32.00 ± 9.36 | 30.33 ± 10.78 | **Assisted Rears** | **Female** | 28.60 ± 10.26 | 30.30 ± 9.51 |
| | **Male** | 23.38 ± 9.54 | 29.44 ± 9.89 | | **Male** | 29.57 ± 14.66 | 24.29 ± 7.02 |
| **Unassisted Rears** | **Female** | 3.10 ± 2.60 | 2.50 ± 0.55 | **Unassisted Rears** | **Female** | 4.10 ± 5.00 | 3.10 ± 2.38 |
| | **Male** | 4.38 ± 5.58 | 3.67 ± 3.16 | | **Male** | 6.29 ± 4.50 | 5.57 ± 6.35 |
| **Total Rears** | **Female** | 35.10 ± 10.42 | 32.83 ± 11.27 | **Total Rears** | **Female** | 32.70 ± 13.52 | 33.20 ± 11.34 |
| | **Male** | 27.77 ± 11.63 | 33.11 ± 11.83 | | **Male** | 35.86 ± 18.69 | 29.86 ± 12.40 |
| **Footfalls** | **Female** | 89.30 ± 26.34 | 85.20 ± 14.69 | **Footfalls** | **Female** | 80.00 ± 30.02 | 94.80 ± 28.79 |
| | **Male** | 81.54 ± 29.08 | 98.22 ± 24.99 | | **Male** | 110.00 ± 38.21 | 98.13 ± 23.47 |

Rearing behavior and footfalls in an open field arena (40.64 x 40.64 x 38.1 cm) modified with a 2 cm elevated wire mesh platform. Rears were categorized as assisted if the rat used forelimbs to brace against side of open field arena. All values presented as mean ± SD. Data for unassisted rears was square root transformed for analysis. Analyzed by 3-way ANOVA with Fisher's LSD multiple comparisons tests, n = 6–13/group. *CIH: Chronic intermittent hypoxia; MT: MitoTEMPOL.*

**No effects of sex or CIH on anxiety-like behavior in an open field.**

We examined anxiety-like behaviors using a large novel open field (60.96 x 60.9 x 38.1 cm) by quantifying the frequency and duration of entries within the center of the large open field. No effects of CIH or sex were observed on anxiety-like behaviors - center entries or center duration (Table 5). However, inhibition of mtOS did decrease open field anxiety-like behaviors, as evidenced by decreased center entries ($F_{1, 60}$=7.434; p = 0.008; $\eta^2$ = 0.110) and center duration ($F_{1, 60}$=7.301; p = 0.009; $\eta^2$ = 0.101; Table 5).

Table 5
Inhibiting mitochondrial oxidative stress increased anxiety-like behavior in an open field arena

| Normoxia | | | | CIH | | | |
|---|---|---|---|---|---|---|---|
| | | Vehicle | MT** | | | Vehicle | MT** |
| **Center Entries** | **Female** | 21.50 ± 7.50 | 15.67 ± 4.59 | **Center Entries** | **Female** | 23.70 ± 13.47 | 16.10 ± 8.89 |
| | **Male** | 22.85 ± 11.20 | 17.00 ± 6.08 | | **Male** | 22.86 ± 8.82 | 16.00 ± 8.89 |
| **Center Duration (s)** | **Female** | 45.80 ± 15.40 | 27.65 ± 6.73 | **Center Duration (s)** | **Female** | 39.74 ± 18.82 | 33.29 ± 24.23 |
| | **Male** | 53.58 ± 23.73 | 42.26 ± 16.80 | | **Male** | 47.63 ± 13.46 | 32.16 ± 15.79 |

Inhibiting mitochondrial oxidative stress reduced center entries and center duration (s) in an open field arena (60.96 x 60.9 x 38.1 cm). No effects of CIH or sex were observed on center entries or center duration. All values presented as mean ± SD. Analyzed by 3-way ANOVA with Fisher's LSD multiple comparisons tests, n = 6–13/group. ANOVA significance for main effect of MT indicated by: ** = $p < 0.01$; Post-hoc significance indicated by unique letters ($p \leq 0.05$). Shared letters are not significantly different. *CIH: Chronic intermittent hypoxia; MT: MitoTEMPOL.*

**CIH increased compulsive behavior in male rats that was prevented by blocking mitochondrial oxidative stress.**

We examined the effects of CIH, sex, and mtOS inhibition on compulsivity using the marble burying task. We observed multiple significant interactions on marble burying between: 1) CIH and sex ($F_{1, 64}$=6.499; p = 0.013; $\eta^2$ = 0.075), 2) CIH and MT ($F_{1, 64}$=5.669; p = 0.020; $\eta^2$ = 0.065), and 3) MT and sex ($F_{1, 64}$=4.437; p = 0.039; $\eta^2$ = 0.051; Fig. 7). CIH increased marble burying in vehicle males ($p \leq 0.05$), which was prevented by blocking mtOS with MT. However, CIH did not affect marble burying in vehicle females. Inhibiting mtOS in normoxic females increased marble burying ($p \leq 0.05$).

## Discussion

The major findings of this study are 1) CIH-induced OS and inflammation are sex- and mtOS-dependent, whereas 2) CIH-induced behavioral changes are dependent on multiple factors that include sex, mtOS, and other mechanisms (Fig. 8). Specifically, we found that CIH increased inflammation and fine motor dysfunction in female rats, which was blocked by inhibiting mtOS. Conversely, CIH increased circulating OS and compulsive behavior in males, which was blocked by inhibiting mtOS. However, we observed no sex differences in CIH-induced recollective memory impairment. Both male and female rats exposed to CIH performed worse than normoxic controls in the novel object recognition task, which was unaffected by inhibition of mtOS.

This study is the first study to examine the effects of CIH on inflammatory cytokines in females, along with the first study to compare sex differences in CIH-induced inflammatory cytokines. We observed that

CIH increased circulating IL-6 levels and IL-6/IL-10 ratio in females. IL-6 is a pleiotrophic cytokine that is associated with many inflammatory diseases [90], whereas IL-10 is an anti-inflammatory cytokine that is linked with downregulating the effects of IL-6 in the immune response [93, 96]. The IL-6/IL-10 ratio indicates the status of pro-inflammatory and anti-inflammatory processes in circulation [95, 96]. Irrespective of CIH, we observed sex differences in cytokine levels. In vehicle rats, normoxic males had higher levels of IL-6 than normoxic females. Few studies have examined sex differences in basal plasma cytokine levels; however, female rats typically have higher levels of IL-6 and TNF-α than males [97, 98]. In our study, we found no sex differences in any other cytokines, which is consistent with other studies examining sex and cytokine levels in plasma [97, 98].

Although prior studies examining the impact of CIH on the immune system have only examined male rodents, they observed increased pro-inflammatory cytokines IL-6, TNF-α, and IL-1β in the circulation in response to CIH [47, 48, 99, 100]. In contrast to these studies, our study found that CIH did not impact IL-6, TNF-α, or IL-1β levels in male rats. This negative finding is consistent with our previous publication that showed no effect of CIH on IL-6 levels (Normoxic males: 558.54 ± 778.63 pg/mL; CIH males: 418.66 ± 551.45 pg/mL; mean ± SD) or TNF-α levels (Normoxic males: 5.16 ± 5.41 pg/mL; CIH males: 3.94 ± 4.03 pg/mL; mean ± SD) [6]. One possible explanation for this discrepancy in CIH-induced cytokine levels is the CIH protocol. Our studies utilized a CIH protocol with an AHI of 10 (10 hypoxic episodes/hr/over 8 hrs/day) to model mild sleep apnea in humans [25, 72]. In contrast, other laboratories used different CIH protocols in male rats that range in AHIs of 30–40 with similar CIH protocol duration (8 hrs/day; 14–21 days) [48, 99, 100], which is consistent with severe sleep apnea in humans [25]. This could indicate that the inflammatory response to CIH in males is dependent on AHI severity.

Similar to our cytokine data, we observed sex differences in circulating oxidized protein levels (AOPP). Consistent with prior reports [6, 49, 50], we found that CIH increased AOPP concentrations in male rats. However, this is the first study to show that CIH-elevated AOPP in male rats was mediated by mtOS. In contrast to male rats, CIH did not increase AOPP in female rats, which is consistent with other studies showing no effects of CIH on circulating OS measures [49, 101]. We also found sex differences in basal circulating OS, in which normoxic female rats had higher OS levels than male rats. This finding is consistent with clinical data wherein women frequently exhibit higher circulating OS than men [35–37].

This study is the first to examine sex differences in CIH-induced behavior displayed by adult rats. We observed distinct sex differences in CIH-induced fine motor function impairment and compulsivity but not in CIH-induced recollective memory impairments. CIH reduced fine motor function only in females, which was blocked by inhibiting mtOS. Since inhibiting mtOS decreased CIH-induced inflammation in females, we propose that a mtOS-inflammation feed-forward mechanism is involved in CIH-induced fine motor impairment in females (Fig. 8A). Indeed, inflammation has been observed to impair motor behaviors [102, 103], which may be related to CIH-induced neuroinflammation in motor behavior-associated brain regions in rats [6, 99].

This is the first study to examine the impact of CIH on marble burying. We observed that CIH increased compulsive behavior in male rats but not female rats. Further, inhibition of mtOS blocked CIH-induced compulsivity in males. Since inhibiting mtOS decreased CIH-induced OS in our males and CIH increases OS damage to brain regions [6, 57, 104] associated with marble burying behavior (e.g., hippocampus, orbitofrontal cortex, striatum) [77, 105, 106], mtOS may be one of the primary mechanisms in CIH-induced compulsivity in male rats (Fig. 8A).

We did not observe any sex differences in CIH-induced recollective memory impairment. Consistent with prior studies showing CIH-induced impairments in recollective memory in male rodents [51–53], we also found that CIH impaired recollective memory in male rats. CIH also decreased recollective memory in females, and our study is the first to examine these relationships. Interestingly, inhibiting mtOS did not prevent the effects of CIH on recollective memory. This indicates that a mtOS-independent mechanism induced by CIH is impairing recollective memory in both sexes (Fig. 8B). Although no prior studies have examined the effect of CIH on recollective memory in females, studies have examined sex differences in recollective memory in response to neurotoxins. These findings on sex differences in recollective memory were equivocal, in which some studies show no sex differences in recollective memory impairment [107, 108] or only males were impaired and not females [107, 109].

CIH had no effects on spatial learning and memory or anxiety-like behavior in either male or female rats. Our findings are consistent with prior reports demonstrating CIH did not impact spatial learning and memory in gonadally intact female mice [59]. However, in contrast to prior studies showing CIH impairment of spatial learning and memory in male rats [47, 54–56], we did not observe any effects of CIH on spatial learning and memory in males. Studies routinely report males exposed to CIH exhibit increased anxiety-like behaviors [52, 57, 58], which was not observed in this study. It should be noted that these prior studies in which CIH induced spatial learning and memory impairments and anxiety-like behaviors were conducted with different CIH protocols (AHI ranged from 40–60) [47, 52, 54–58] compared to our CIH protocol (AHI = 10). Indeed, a study using a CIH protocol with an AHI of 20 found no effect of CIH on spatial learning and memory in male rats [110], and another study using an AHI of 15 found no effects on anxiety-like behavior in the open field in male and female mice [59]. These findings indicate that CIH protocols with > 20 AHI is necessary to induce spatial learning and memory impairments and anxiety-like behaviors.

We also observed sex differences in learning and motor behaviors exhibited by our normoxic rats. Consistent to prior reports showing females exhibit greater recollective memory than males [111–113], we also found that females exhibited better recollective memory (shorter latency to novel object) than males. In addition to better recollective memory, females exhibited greater spatial learning ability (lower WLI scores) than males. Prior studies have typically shown either male biases [71, 111, 114] or no sex differences in spatial learning via Morris water maze [114, 115], which is in contrast to our findings. Notably, these studies did not use a WLI [71, 111, 114] or weighted both males and females against male control values [115]. As our WLI scores were weighted against within-sex and within-experiment control values, this may have increased the sensitivity of our measure to detect previously unobserved sex differences [88, 89]. Neither unweighted LI scores nor latency to platform during the probe trial detected significant sex differences, further underscoring the importance of using WLI scores as a measure of Morris water maze spatial learning ability [88, 89]. In addition, our Morris water maze protocol includes a pre-training day on day 1, which has been previously observed to improve female performance [116, 117]. Consistent with prior reports, female rats showed greater locomotor activity than male rats in a large novel open field arena (60.96 x 60.9 x 38.1 cm) and no sex differences in a smaller novel open field (40.64 x 40.64 x 38.1 cm)[46, 118]. Unlike these findings, we did not observe any sex differences in marble burying behavior in normoxic rats. The data on sex differences in marble burying is equivocal, with some groups finding either no sex differences [119, 120], a female bias [121], or a male bias [71, 122].

## Limitations

Although our study has many strengths ranging from examining a broad spectrum of circulating markers of inflammation and OS and multiple behavioral domains, there are some limitations. Due to limited plasma, we were unable to perform analysis of cytokines in normoxic rats treated with MT. Additionally, we did not assess estrous cycle in our female rats. It is probable that the variability in the female data is related to estrous cycle, as many of the outcomes measured in our study could be influenced by estrogen status [123–125]. However, we did not want to introduce a stress response by conducting vaginal smears [126], nor introduce a variable that could not be conducted on the male rats. Lastly, we did not determine the brain concentrations of MT. However, our data showing behavior responses to MT, data showing that MT has similar membrane permeability to TEMPOL [68], and data that TEMPOL penetrates the blood-brain-barrier [67] indicates that MT has actions at the level of the brain.

## Perspectives and significance

Our data indicates that mild CIH (AHI = 10) can have significant impacts on multiple domains, such as circulating OS and inflammation, recollective memory, fine motor function and compulsivity. Furthermore, most of these CIH effects are sex- and mtOS-dependent with the exception of recollective memory impairment. This is of concern, as patients with sleep apnea with AHIs < 15 are classified as having mild OSA [14, 16, 18], and therefore may not receive treatment due to fewer reported quality of life impairments [4, 127, 128]. This lack of treatment for mild OSA can affect women more than men, as women are more likely to present with mild OSA or be underdiagnosed with OSA [4, 5]. We also show that many of the sex-dependent effects of CIH are mediated through mtOS, which may be a plausible target for therapeutics aimed at 15–40% of OSA patients that are unable to be effectively treated with CPAP machines [20–22].

## Declarations

#### Ethics approval:

This study was performed under IACUC protocol approved by the University of North Texas Health Science Center.

**Consent for publication:**

Not applicable.

**Availability of data and materials:**

The datasets used and/or analyzed during the current study are available from the corresponding author upon reasonable request.

**Competing interests:**

The authors declare that they have no competing interests.

**Funding:**

This study was supported by NIH R01 NS0091359 and UNTHSC Seed grant funding to RLC, AHA 22PRE-900431 to JJG, AHA 22POST-903250 to JLB, and NIH T32 AG020494 to SM.

**Author's contributions:**

SM conducted experiments, collected and processed tissue, analyzed data, and wrote the manuscript. ENW, JLB, and JJG conducted experiments and assisted with tissue collection and processing. RLC designed the study, analyzed data, interpreted the findings, and edited the manuscript. RLC and SM were major contributors in writing the manuscript. All authors read and approved the final manuscript.

**Acknowledgements:**

We would like to acknowledge UNTHSC Office of Vice President of Research for supporting these studies.

## Figures

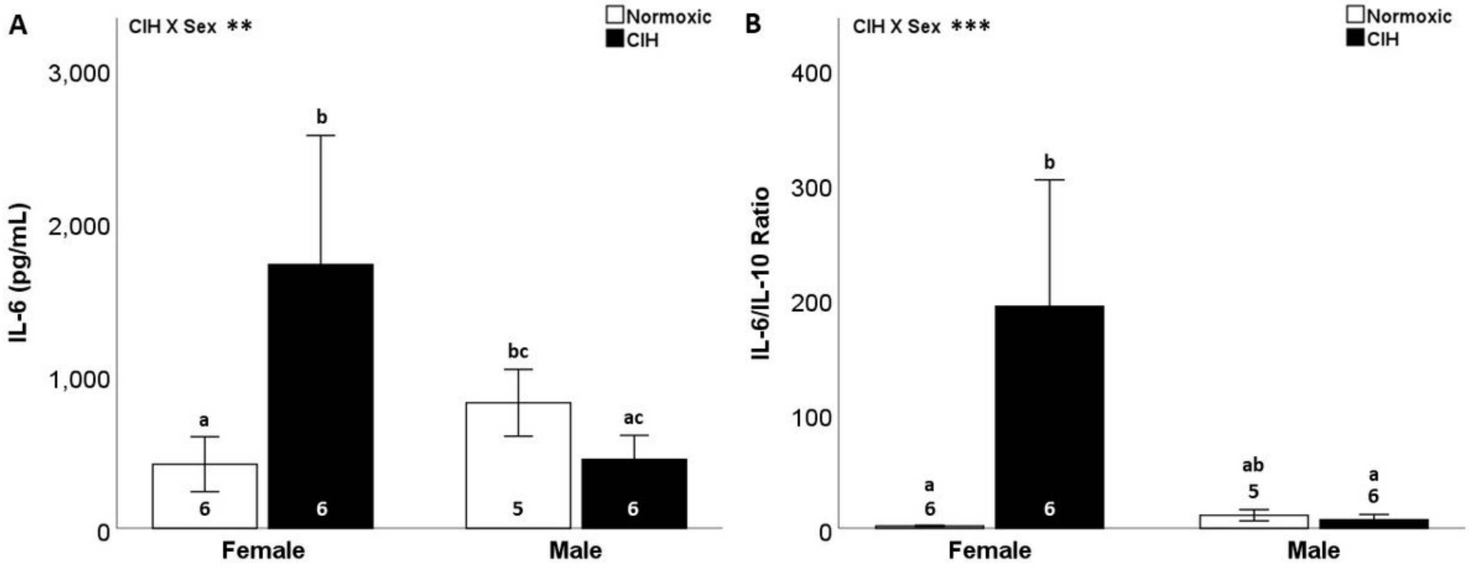

### Figure 1

**Impact of sex and CIH on circulating inflammatory cytokines.** CIH only increased IL-6 levels in female rats (A). CIH only increased IL-6/IL-10 ratio in females (B). Data for inflammatory cytokines was log transformed for analysis. Analyzed by 2-way ANOVA with Fisher's LSD multiple comparisons tests. ANOVA significance indicated by: ** = $p \leq 0.01$; *** = $p < 0.001$; Post-hoc significance indicated by unique letters ($p \leq 0.05$). Shared letters are not significantly different. *CIH: Chronic intermittent hypoxia.*

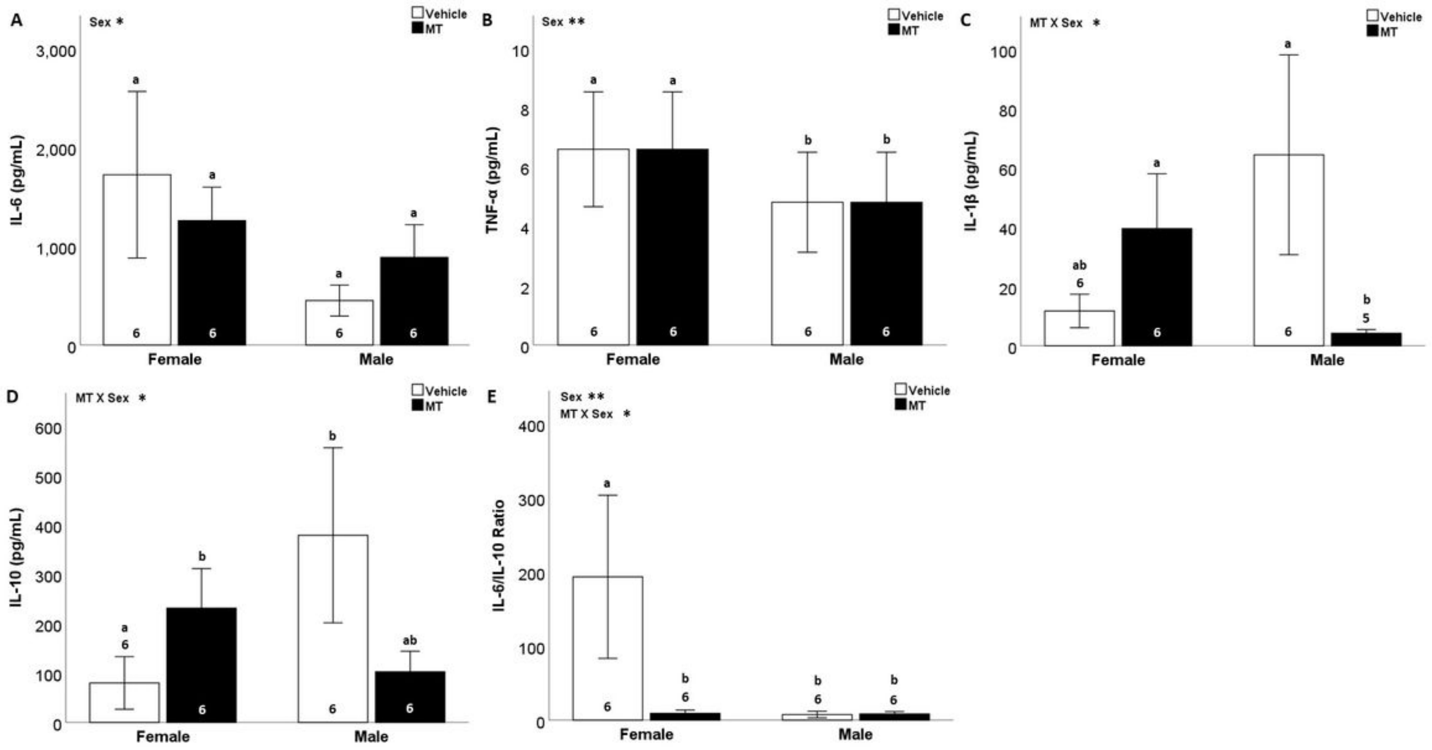

### Figure 2

**Impact of inhibition of mitochondrial oxidative stress on circulating inflammatory cytokines in CIH rats.** Female rats had higher IL-6 levels than males, regardless of MT (A). Females had higher levels of TNF-α than males, regardless of MT (B). IL-1β levels were only reduced by MT in males (C). IL-10 levels were increased by MT in females (D). IL-10 levels were higher in vehicle males than vehicle females (D). IL-6/IL-10 ratio was only elevated in vehicle females, and lowered by MT (E). Data for inflammatory cytokines was log transformed for analysis. Analyzed by 2-way ANOVA with Fisher's LSD multiple comparisons tests. ANOVA significance indicated by: * = p≤0.05; ** = p≤0.01; *** = p<0.001; Post-hoc significance indicated by unique letters (p≤0.05). Shared letters are not significantly different. *CIH: Chronic intermittent hypoxia; MT: MitoTEMPOL.*

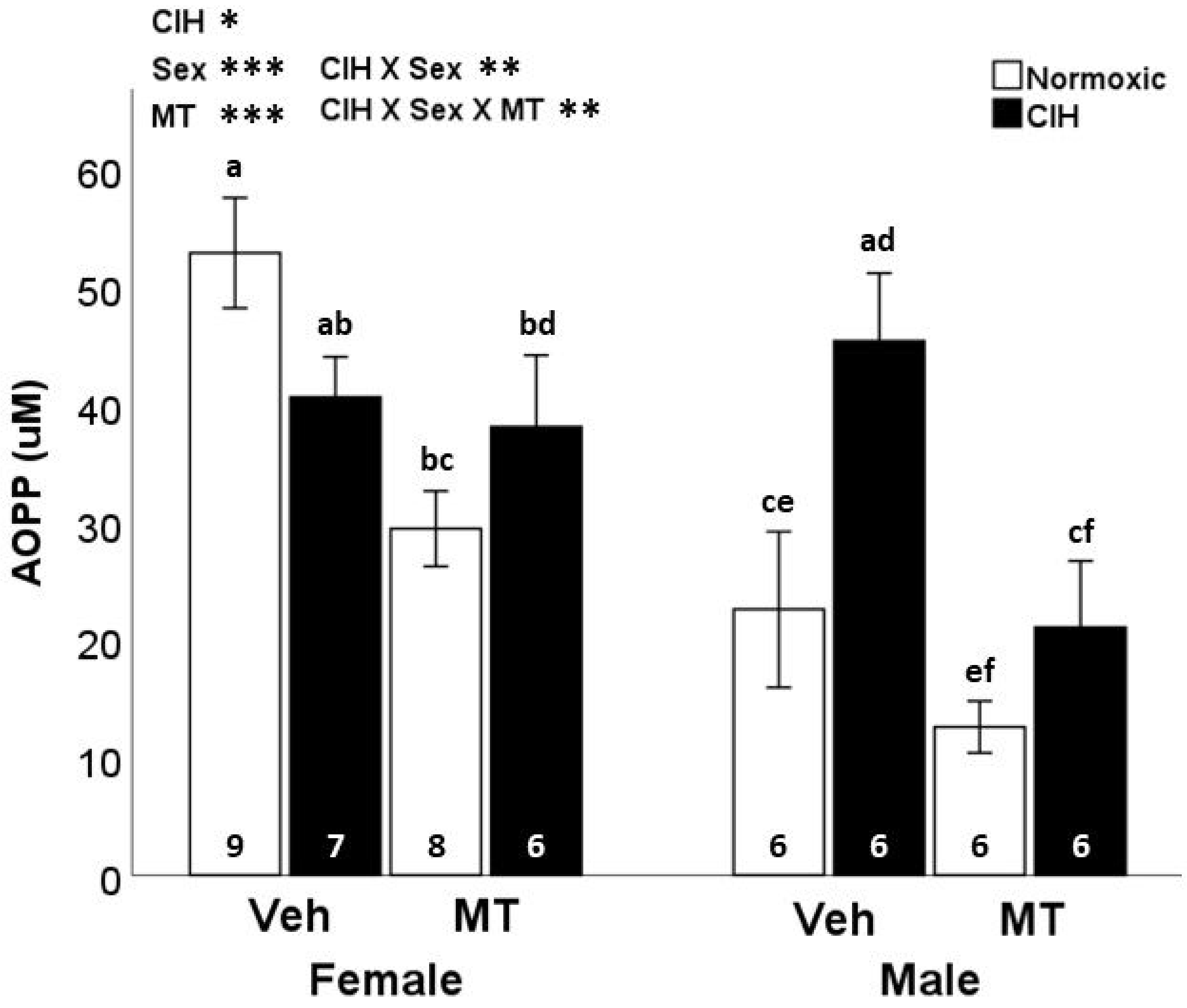

Figure 3

**Circulating oxidative stress.** AOPP concentrations were dependent on CIH, sex, and MT. In female rats, normoxic females had the highest AOPP concentrations, and these were reduced by MT. CIH also reduced AOPP concentrations in vehicle females, but did not affect AOPP concentrations in MT females. AOPP concentrations were higher in females than males. In vehicle males, CIH increased AOPP concentrations, and this effect was blocked by MT. Analyzed by 3-way ANOVA with Fisher's LSD multiple comparisons tests. ANOVA significance indicated by: * = $p \leq 0.05$; ** = $p \leq 0.01$; *** = $p < 0.001$; Post-hoc significance indicated by unique letters ($p \leq 0.05$). Shared letters are not significantly different. *AOPP: Advanced oxidation protein products; CIH: Chronic intermittent hypoxia; MT: MitoTEMPOL.*

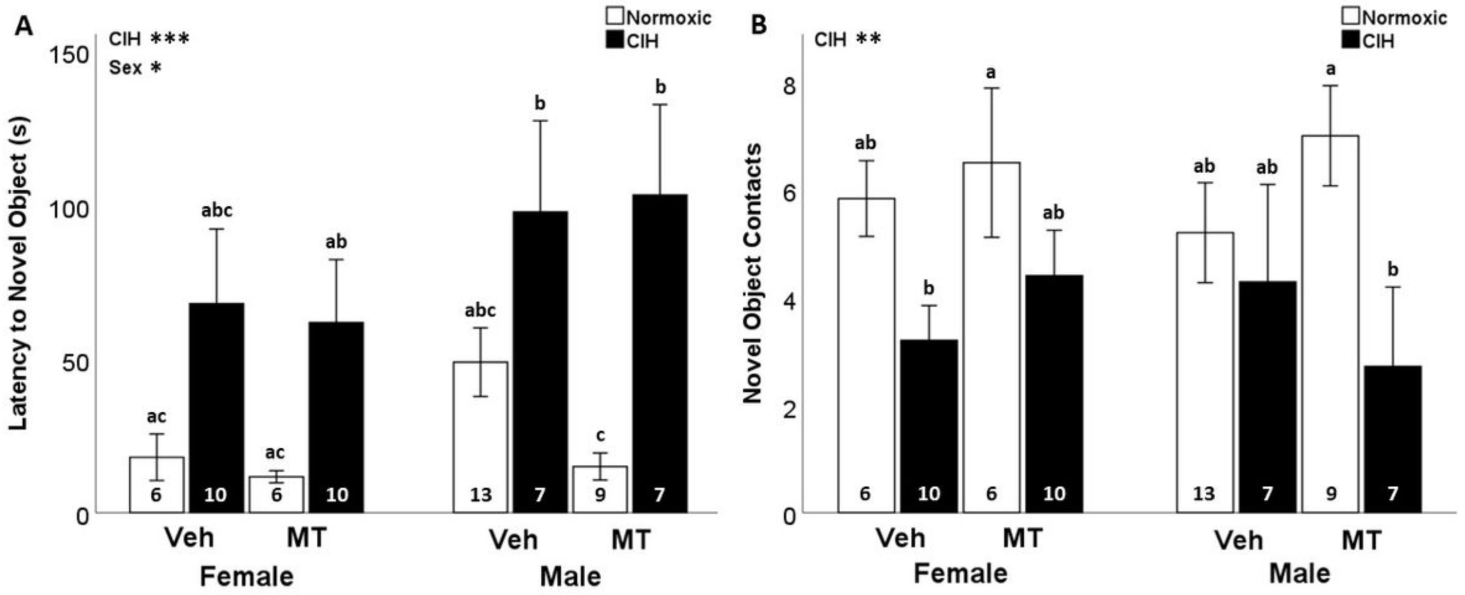

### Figure 4

**Recollective memory.** CIH impaired recollective memory in the novel object recognition task by increasing latency to the novel object (A) and decreasing novel object contacts (B). Female rats had shorter latency to the novel object than males (A). No effect of sex was observed on novel object contacts (B). No effect of MT was observed on recollective memory (A, B). Data for novel object latency was log transformed for analysis. Analyzed by 3-way ANOVA with Fisher's LSD multiple comparisons tests. ANOVA significance indicated by: * = $p \leq 0.05$; ** = $p \leq 0.01$; *** = $p < 0.001$; Post-hoc significance indicated by unique letters ($p \leq 0.05$). Shared letters are not statistically significant. *CIH: Chronic intermittent hypoxia; MT: MitoTEMPOL.*

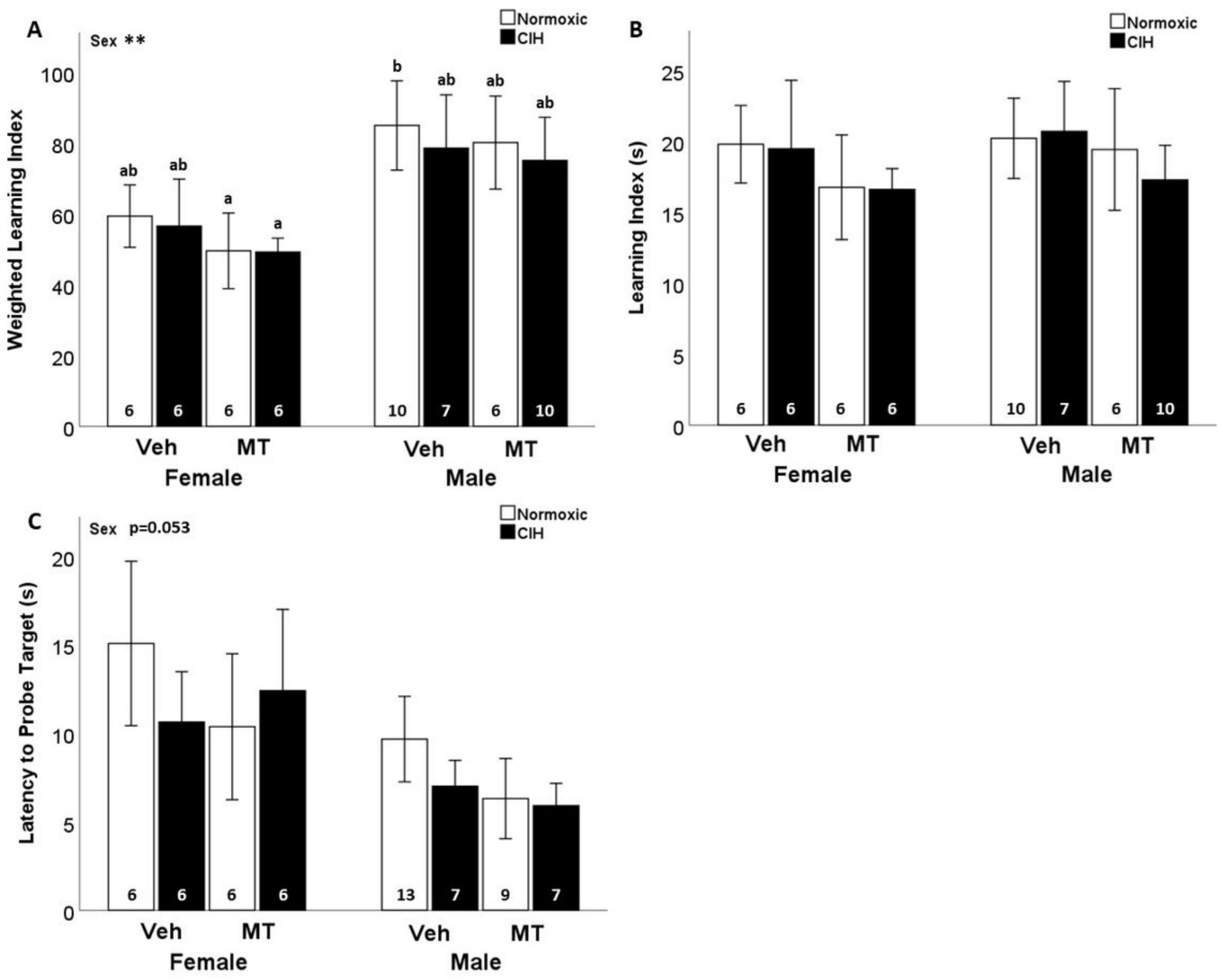

### Figure 5

**Spatial learning.** Male rats exhibited greater spatial learning in the Morris water maze than females, with higher weighted learning index scores. No effects of CIH or MT were observed on weighted learning index scores. Analyzed by 3-way ANOVA with Fisher's LSD multiple comparisons tests. ANOVA significance indicated by: ** = p≤0.01; Post-hoc significance indicated by unique letters (p≤0.05). Shared letters are not significantly different. *CIH: Chronic intermittent hypoxia; MT: MitoTEMPOL.*

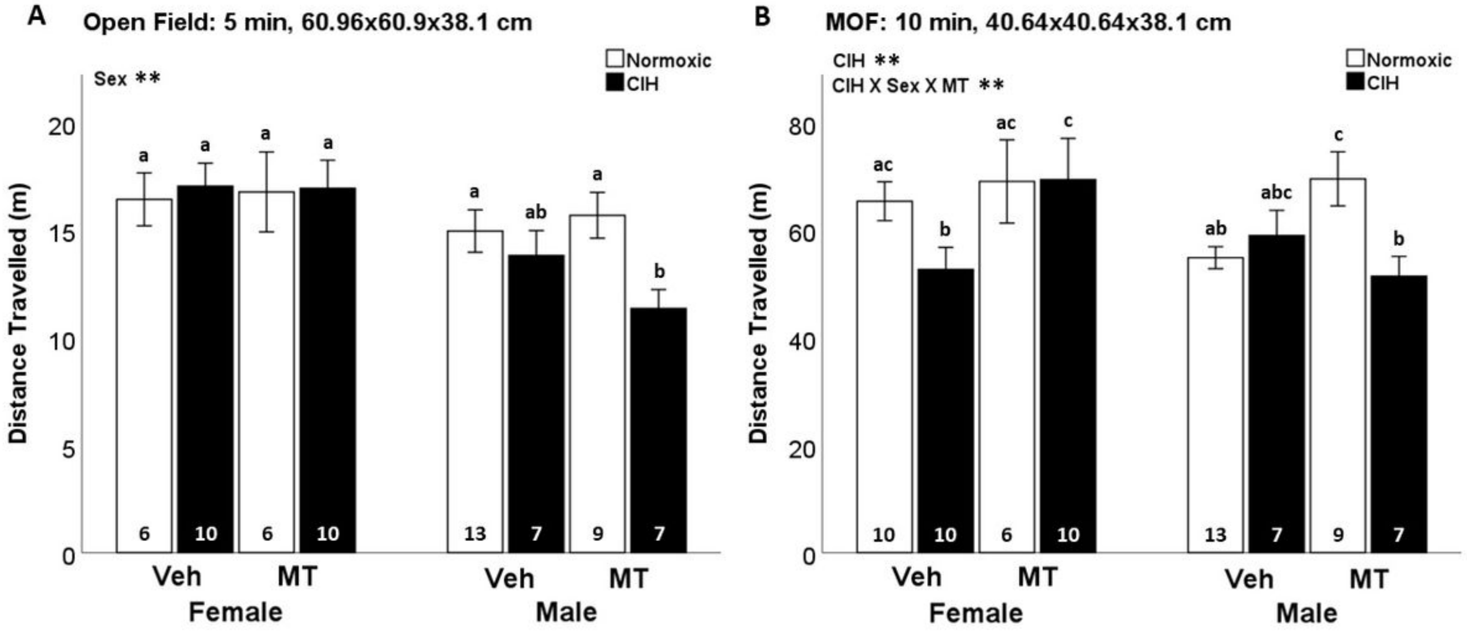

### Figure 6

**Motor function.** Female rats traveled farther in the open field than males (A). No effect of CIH or MT was observed in the open field (A). Distance traveled in the MOF was dependent on CIH, sex, and MT (B). CIH decreased MOF distance traveled in vehicle females, which was prevented by MT (B). CIH MT females had elevated MOF distance traveled compared to both normoxic vehicle males, and CIH MT males (B). In normoxic males, MT increased MOF distance traveled, but this effect was not seen in CIH males (B). Data for MOF distance traveled was log transformed for analysis. Analyzed by 3-way ANOVA with Fisher's LSD multiple comparisons tests. ANOVA significance indicated by: ** = $p \leq 0.01$; Post-hoc significance indicated by unique letters ($p \leq 0.05$). Shared letters are not significantly different. *CIH: Chronic intermittent hypoxia; MOF: Modified open field; MT: MitoTEMPOL.*

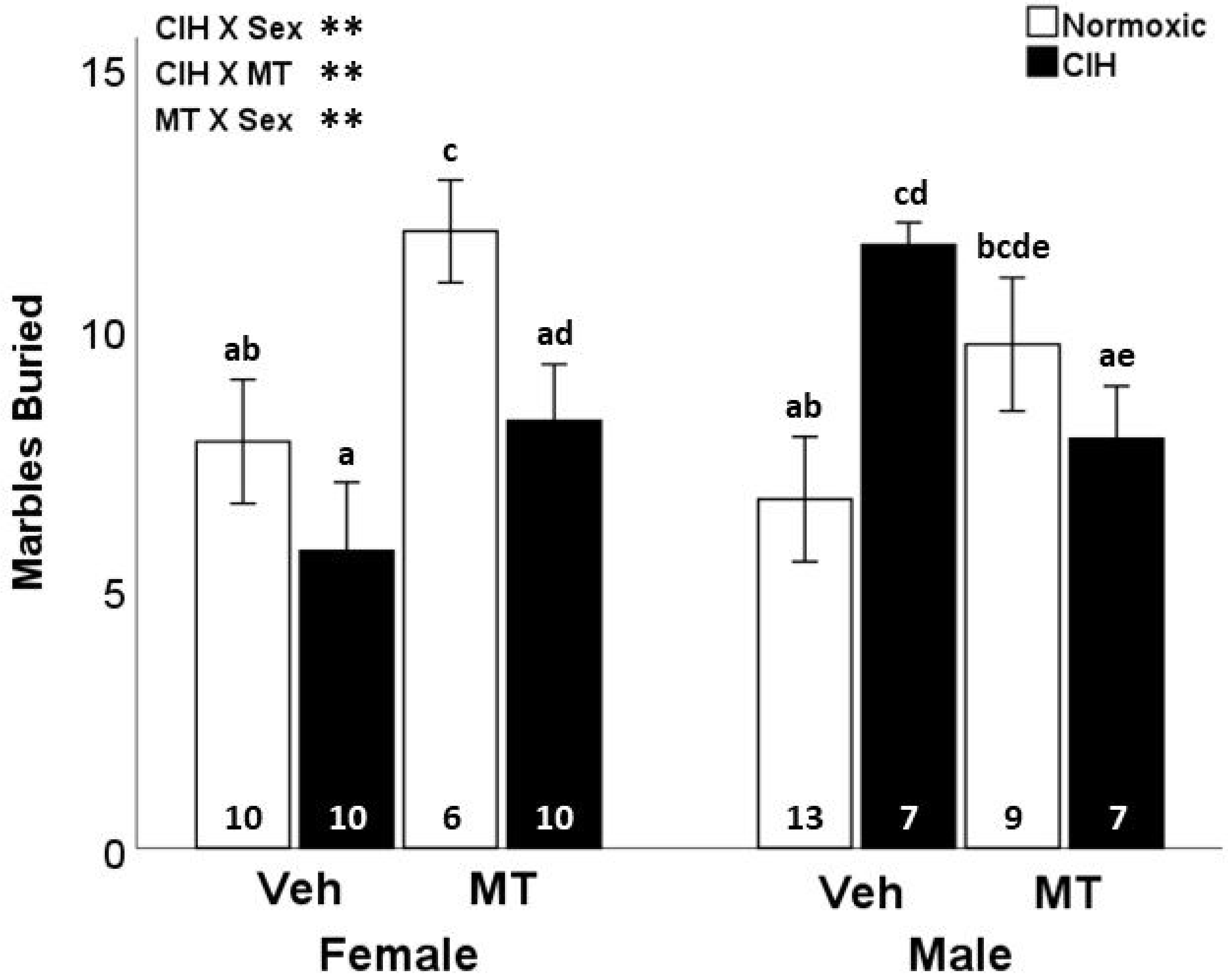

Figure 7

**Compulsive behaviors.** CIH increased marble burying in vehicle male rats, but not in vehicle females. MT prevented CIH from increasing marbles buried in males. In MT females, CIH decreased marble burying, with no effect in MT males. MT increased marble burying in normoxic females, but not in normoxic males. Analyzed by 3-way ANOVA with Fisher's LSD multiple comparisons tests. ANOVA significance indicated by: ** = p≤0.01; Post-hoc significance indicated by unique letters (p≤0.05). Shared letters are not significantly different. *CIH: Chronic intermittent hypoxia; MT: MitoTEMPOL.*

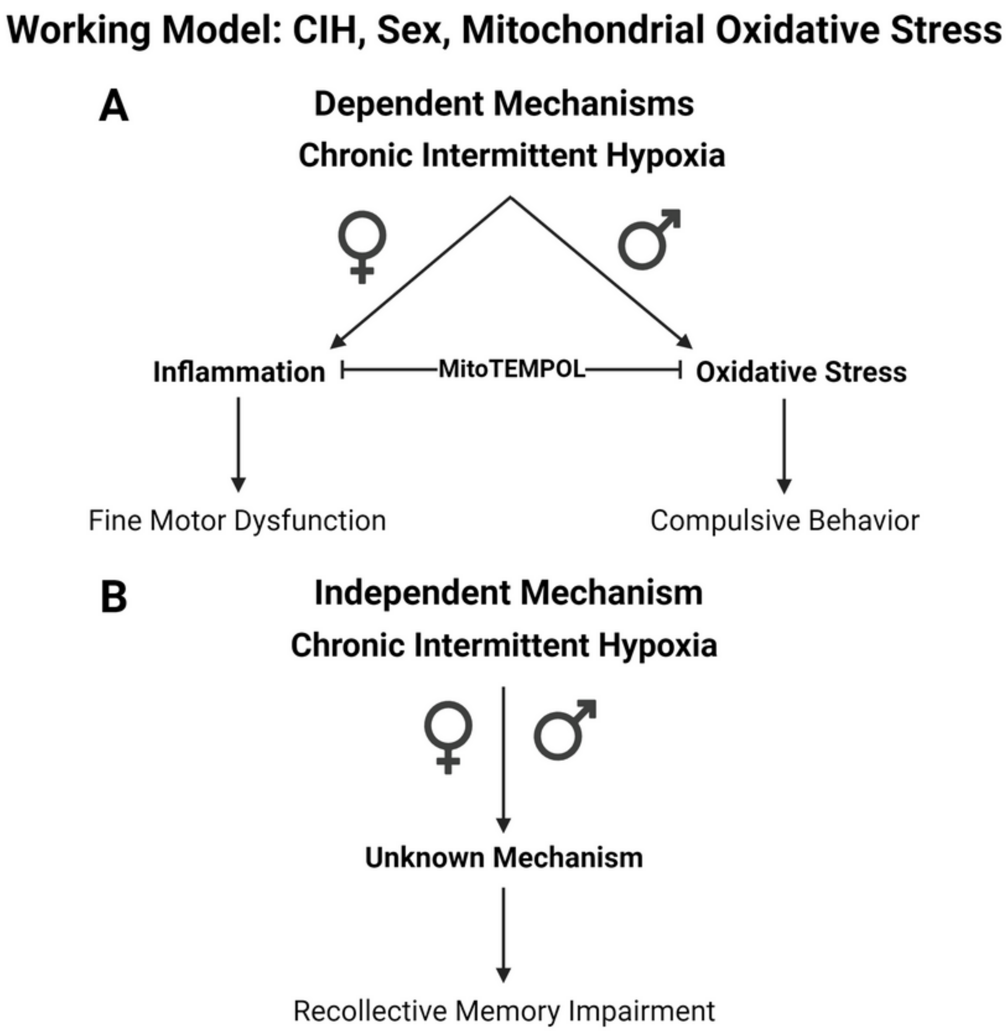

Figure 8

**Working model: CIH, sex, mitochondrial oxidative stress.** Model of CIH induced impairments that are mitochondrial OS-dependent (A) and mitochondrial OS-independent (B). Created with Biorender.com. *CIH: Chronic intermittent hypoxia.*
